# Zebrafish—A Model Organism for Studying the Neurobiological Mechanisms Underlying Cognitive Brain Aging and Use of Potential Interventions

**DOI:** 10.3389/fcell.2018.00135

**Published:** 2018-11-01

**Authors:** Michelle M. Adams, Hulusi Kafaligonul

**Affiliations:** ^1^Interdisciplinary Neuroscience Program, Aysel Sabuncu Brain Research Center, Bilkent University, Ankara, Turkey; ^2^Department of Psychology, Bilkent University, Ankara, Turkey; ^3^National Nanotechnology Research Center (UNAM), Bilkent University, Ankara, Turkey; ^4^Department of Molecular Biology and Genetics Department Zebrafish Facility, Bilkent University, Ankara, Turkey; ^5^National Magnetic Resonance Research Center (UMRAM), Aysel Sabuncu Brain Research Center, Bilkent University, Ankara, Turkey

**Keywords:** aging, cognition and perception, behavior, neurobiological alterations, interventions, dietary restriction

Classically, the zebrafish model organism has been used to elucidate the genetic and cellular mechanisms related to development since the embryo forms and grows externally following fertilization. This provides insight into the genetic control of developmental processes in humans because their genomes are similar. Also, unlike other animal models, the genes of zebrafish can be manipulated quite easily by using reverse genetic screens tools such as morpholinos, which transiently silence target genes of interest or systems such as the transposon-mediated insertional mutagenesis or CRISPR-Cas9. Moreover, one pair of fish will provide up to 300 offspring, which means that if there is a gene of interest that is manipulated, then it can be transmitted to a large population of fish. What is beginning to emerge is that similar to other mammals, adult zebrafish have an integrated nervous system, which is proposed to contain homologous brain structures to those found in humans, as well as equivalent cellular and synaptic structure and function. Moreover, like humans, zebrafish exhibit age-related declines in cognitive functions, and a convergence of evidence has indicated that subtle changes in cellular and synaptic integrity underlie these changes. Therefore, the zebrafish is a powerful model organism for studying the neurobiological consequences of aging-related behavioral and biological changes, which offers the potential to identify possible interventions that would promote healthy aging. In what follows, we present and discuss recent findings and advances along these directions.

## Behavioral tasks and abilities altered in aged zebrafish

The zebrafish is a promising model for studying age-related changes in cognition and perception. Early behavioral studies date back to 1960s and the characterization of zebrafish behavior has accelerated since 2000 (Kalueff et al., 2013). They have been suggested to reflect the evolutionarily conserved nature of many behaviors and to resemble those of other species (Kalueff et al., 2014; Stewart et al., 2014; Orger and de Polavieja, 2017). A rich repertoire of behavioral phenotypes has been identified for cognitive functioning, perceptual processes, and associated disorders (Stewart and Kalueff, 2012). Using different behavioral assays (e.g., inter- and intra-trial habituation, T-maze, conditioned place preference paradigms), previous studies indicated that zebrafish have both simple and relatively complex forms of learning, and also display good performance on cognitive tasks dependent on short-term and long-term memory (Blaser and Vira, 2014; Gerlai, 2016). There is also growing interest in other aspects of zebrafish behavior which significantly depend on perception, low-level discrimination, and sensitivity (Neuhauss, 2010). For instance, the basic components of the zebrafish visual system, the visual processing hierarchy, and pathways are similar to those commonly found in other species (Bilotta and Saszik, 2001). In particular, most of the previous research evaluated visual motion perception and sensitivity through optomotor response and/or optokinetic reflexive eye movements. These behavioral studies point to qualitatively similar visual acuity and contrast sensitivity functions for zebrafish (Rinner et al., 2005; Haug et al., 2010; Tappeiner et al., 2012). It has also been shown that zebrafish perceive first- and second-order motion. They also experience motion illusions commonly used in studies on human vision such as reverse-phi illusion, motion aftereffect, and rotating snakes illusion (Orger et al., 2000; Gori et al., 2014; Najafian et al., 2014). Within the context of visual motion, these studies provide behavioral evidence that mechanisms and principles similar to those of humans and other species underlie zebrafish sensory processing and associated behavior.

Characterizing aging-related changes in zebrafish behavior has important implications for our understanding of cognition and perception. First, aging-related changes in cognition are a part of the normal aging process and common in all the species. Monitoring age-dependent changes in cognition and perception is difficult to perform on the same human subject throughout life. Due to their short lifespan, behavioral assays and paradigms developed, zebrafish provides an ideal model to study cognitive and perceptual performance during aging. Second, when these behavioral studies are combined with already developed molecular and genetic tools on this aging model, we will also have a deeper understanding on the functional links between key synaptic targets, cognition, and perception during neural aging. Previous studies report significant declines in learning and memory in aged zebrafish. Typically, old zebrafish have less performance on tasks relevant with associative learning, avoidance, spatial learning and working memory (Yu et al., 2006; Arey and Murphy, 2017; Brock et al., 2017). Compared to wild-types, mutants with impaired acetylcholinesterase function had better performance in spatial learning, entrainment and increased rate of learning (Yu et al., 2006; Parker et al., 2015). These findings suggest that cholinergic signaling may also play a role in age-related cognitive decline. In terms of perceptual performance, there are studies comparing larvae and adult zebrafish. However, we have limited knowledge on how perceptual performance (and thus perception and sensitivity) changes during neural aging. A challenge for the future is to characterize aging-related changes in perceptual performance and sensitivity of adult zebrafish. As mentioned above, we consider that such studies can provide comprehensive information not only on perception and behavior in general (Owsley, 2016) but also on the cellular and molecular mechanisms underlying specific aspects (e.g., motion) of perception and sensitivity.

## Aging-related neurobiological alterations

Understanding the cellular mechanisms that underlie cognitive decline is important for determining sites of actions for possible interventions that could ameliorate alterations in cognitive function. Early reports indicated that age-related cognitive decline was due to significant cell (Brody, 1955; Devaney and Johnson, 1980; Henderson et al., 1980) and synapse loss (Geinisman et al., 1977; Bondareff, 1979; Curcio and Hinds, 1983; Haug and Eggers, 1991; Shi et al., 2005). However, it has become well accepted that significant cell (Haug and Eggers, 1991; Rapp and Gallagher, 1996; Rasmussen et al., 1996; Peters et al., 1998) and synapse loss does not occur in conjunction with normal aging-related declines in cognitive capacities (Poe et al., 2001; Newton et al., 2007; Shi et al., 2007). Therefore, research studies have been designed at examining markers of cellular and synaptic integrity during the aging process, such as altered neurogenesis rates (Kempermann et al., 1998, Luo et al., 2006) and the levels of key excitatory and inhibitory pre- and post-synaptic proteins (Newton et al., 2007; Shi et al., 2007; Adams et al., 2008), since subtle changes in cellular and synaptic functions likely underlie the aging-related declines in cognitive abilities. Moreover, examining key molecular targets that control these processes will increase our understanding of the cellular and synaptic regulation of behavior across the lifespan.

While these aging-related changes in cellular and synaptic processes could be examined in many different animal species, the zebrafish model organism is well-adapted to studying the cellular and molecular changes with aging because they have similar patterns as mammals with regards to the cellular aging process. Zebrafish on average live approximately three to five years and share a similar genome with humans (Kishi et al., 2003; Howe et al., 2013). Moreover, senescence-associated ß-galactosidase, which is a biomarker of aging, increases with advancing age in zebrafish, and this cellular alteration has been described in humans as well (Kishi et al., 2003; Arslan-Ergul et al., 2016). Finally, zebrafish have continued neurogenesis even into late adulthood (Kizil et al., 2012; Schmidt et al., 2013), they express key excitatory and inhibitory pre- and post-synaptic proteins (Karoglu et al., 2017), and classical cellular synaptic plasticity (i.e., long-term potentiation) is found in their brains (Nam et al., 2004). Recent work in the zebrafish brain has demonstrated that there are age-related declines in genes related to cellular and synaptic structure and growth (Arslan-Ergul and Adams, 2014), neurogenesis (Edelmann et al., 2013; Arslan-Ergul et al., 2016), and synaptic alterations (Arslan-Ergul et al., 2016; Karoglu et al., 2017). Interestingly, as has been shown in mammals, these changes depend on the gender of the animal (Arslan-Ergul and Adams, 2014; Karoglu et al., 2017), and the data are in good agreement with those showing sexually-dimorphic patterns published in young zebrafish brains (Ampatzis et al., 2012). Taken together, these findings indicate that the zebrafish is an appropriate model to study the effects of cellular and synaptic aging and its relationship to cognitive decline.

## Use of interventions to alter aging-related processes

A major goal of research related to elucidating the altered cellular and synaptic processes that underlie cognitive aging is to determine possible interventions to restore youthful cellular and synaptic function. As was mentioned previously, mutant zebrafish with lower levels of acetylcholinesterase had better performance in spatial learning, entrainment, and increased rate of learning (Yu et al., 2006; Parker et al., 2015). Therefore, these animals likely have a more youthful cellular and synaptic profile as compared to their wild-type counterparts. Currently, we are investigating this possibility and our data suggest that genetic manipulation of the cholinergic system alters the course of aging-related changes in the synaptic protein levels. We have demonstrated that at old ages as compared to their wild-type siblings, mutants have higher levels of synaptophysin, which is an indicator of presynaptic integrity, and gephyrin, a component of post-synaptic inhibitory transmission, and interestingly these changes are gender-dependent (Karoglu et al., 2018). If we can determine the cellular and synaptic profile of these mutants and how they relate to cognitive aging, it would provide potential targets for drug development to ameliorate the effects of cognitive decline.

Another potential intervention with promise is dietary restriction (DR), which is the only non-genetic intervention that reliably increases both lifespan and healthspan. Numerous studies have shown that a lifelong reduction in caloric intake from *ad libitum* levels increases lifespan (Roth et al., 2001; Lin et al., 2002; Colman et al., 2009). Additionally, DR increases neuronal proliferation and survival (Lee et al., 2002; Kitamura et al., 2006; Park and Lee, 2011; Park et al., 2013). We applied a short-term DR of 10 weeks and observed that this treatment did not prevent an age-related decline in cell proliferation but altered the telomere lengths of these neuronal cells (Arslan-Ergul et al., 2016), thereby DR exerted positive effects by subtly altering the cell cycle dynamics of these neurons. We have tested the timing and duration of short-term DR and a potential DR-mimetic, rapamycin, as the positive effects of DR are thought to be modulating the mammalian target of rapamycin signaling pathway. Our data indicate that a longer duration of both DR and its mimetic is more effective on aging-related changes in synaptic protein levels and transcripts, which might reflect a conserved mechanism of the beneficial effects of DR and rapamycin on life- and healthspan (Celebi-Birand et al., 2018). These studies also have the potential to provide suitable therapeutic targets around which drug development can proceed for ameliorating the devastating effects of cognitive decline.

## Conclusions

The zebrafish is clearly a powerful model organism that can be used to understand the aging-related changes in both cognition and the underlying cellular and molecular processes. As previously mentioned, zebrafish exhibit characteristics that are similar to humans, as well as other mammals, including the fact that these animals age gradually, and they demonstrate aging-related changes across both cognitive and neurobiological spectrums. It clear that both genetic and non-genetic interventions can be applied to alter the course of the aging process and provide potential drug targets that could be manipulated to ameliorate age-related cognitive declines. Therefore, this model will help researchers elucidate the biological mechanisms that underlie aging-related cognitive decline.

## Author contributions

All authors listed have made a substantial, direct and intellectual contribution to the work, and approved it for publication.

### Conflict of interest statement

The authors declare that the research was conducted in the absence of any commercial or financial relationships that could be construed as a potential conflict of interest.
